# Plasma volume, cell volume, total blood volume and F factor in the tree shrew

**DOI:** 10.1371/journal.pone.0234835

**Published:** 2020-09-03

**Authors:** Wei Xia, Zong-jian Huang, Zhao-liang Guo, Yi-wei Feng, Chao-yin Zhang, Guang-yao He, An-zhou Tang

**Affiliations:** 1 Department of Otorhinolaryngology Head and Neck Surgery, The First Affiliated Hospital of Guangxi Medical University, Nanning, Guangxi, China; 2 Key Laboratory of Early Prevention and Treatment for Regional High Frequency Tumor, Ministry of Education, Nanning, Guangxi, China; Kunming Institute of Zoology, Chinese Academy of Sciences, CHINA

## Abstract

In this study, the physiological values of volumes of plasma, cells, total blood and the F blood factors were identified in 24 adult tree shrews (Tupaia belangeri; 12 male and 12 female; average BW of 123.9±19.19 g). The two-compartment model method of Evans Blue dye was used to obtain the plasma volume and the venous hematocrit was measured by microhematocrit method. To establish the relationship between body weight (BW) and blood volume of tree shrews, We performed linear fitting for these two datasets. Results were analyzed according to gender and weight (<120g vs.>120g). Statistical significance was assessed using the unpaired student t test and one-way ANOVA. The average volumes per 100g body weight of plasma, red blood cell (RBC) and total blood were 5.42±0.543, 3.24±0.445, and 8.66±0.680ml respectively. The mean body hematocrit, cardiac hematocrit, jugular vein hematocrit, femoral vein hematocrit, and tail vein hematocrit was 37.43±4.096, 39.72±3.219, 43.04±4.717, 40.84±3.041, and 38.71±3.442% respectively. The F cardiac was 0.94±0.072, F jugular vein 0.88±0.118, F femoral vein 0.92±0.111, and the F tail vein 0.97±0.117. Blood volume (ml) was 85.89103×BW (kg). This is the first study to provide the parameters of plasma volume, cell volume, total blood volume and F factor and a baseline for future research on blood physiology of tree shrews.

## Introduction

The tree shrew is a small, squirrel-like mammal with an adult weight of 120 ~ 150g, mainly distributed in southeast Asia. It used to be considered a lower primate because of its unique relationship to primates [1,2]. Therefore, it is considered as an excellent animal model and is widely used in biomedical research [3–6]. Blood volume is an important physiological index [7], operations involving blood sampling of tree shrews need to consider blood volume as one of the parameters measured. However, there are currently no reports regarding the blood volume on tree shrews.

Blood volume is the sum of the blood cell volume and the plasma volume in circulatory system [8]. Theoretically, the accurate blood volume value can be obtained by labeling the plasma and red blood cells (RBCs) simultaneously and then measuring their volumes. Practically, the blood volume of small-bodied animals is small, while large amount of blood is required for the double labeling experimental method. Excessive blood loss in small animals may cause changes in hemodynamics and blood composition. This might affect the measurement results, and even lead to the death of animals and the termination of the experiment [9]. Therefore, for small-bodied experimental animals, it is appropriate to measure the plasma and RBC volumes, and to calculate the blood volume in combination with the hematocrit.

Although it is generally believed that the radionuclide labeling method can provide accurate measurement results [10], it is expensive and have detrimental effects to the environment and the health of subject animals. The Evans Blue (T-1824) dye method is cheaper and avoids radionuclide radioactivity making it safer to the environment and the health of the subjects. As far as plasma measurement is concerned, studies have shown that the result of labeling serum albumin with iodine 131 (131I-labelled) is consistent with Evans Blue dye method [7,9]. Evans Blue dye method has been widely used in the measurement of plasma volume in animals [9,11–15] and humans [16–21].

The accuracy of the Evans Blue dye method for measuring plasma volume is influenced by the uniform mixing time of dye in the blood and the dye leakage rate to the blood vessels [17,22]. In larger experimental animals, log linear method can be used to overcome this difficulty by pushing the dye mixing curve to zero point (at the time of injection) through continuous blood sampling. However, for small experimental animals, repeated blood collection may affect the total blood volume, making the results more inaccurate. Therefore, most previous studies adopted the single point method to draw blood in small animals 5–10 minutes after the dye injection [9,12–15]. Richalet et al. created the two-compartment model for the measurement of plasma capacity in small experimental animals using the Evans blue dye method [20]. Compared with the log linear method and the single point method, the two-compartment model gave more accurate results and evaluated the dye leakage rate simultaneously [20].

No studies concerning hematocrit in tree shrews have been reported as same as blood volume. The mean hematocrit of the whole body is usually smaller than that of blood samples collected from veins or arteries [9,24]. Besides, the hematocrit of different blood sampling sites varies because of the influence of hemodynamics [9,23–25]. Therefore, the mean body hematocrit/vein hematocrit (F factor) should be correct to ensure accurate results are obtained when using blood cell volume or plasma volume to estimate the total blood volume. This study investigated the blood volume of tree shrews using the two-compartment model, calculated the F factor of venous blood in different sites of tree shrews, to provide reference for the related research on blood volume of tree shrews in the future.

## Materials and methods

### Ethics statement

The protocols used in this study were approved by the Animal Ethics Review Committee of Guangxi Medical University (approval number: 201911062). The experimental protocols strictly followed the Guiding Principles for the Use and Care of Experimental Animals issued by the Ministry of Science and Technology of China.

### Animals

A total of 24 clean-grade tree shrews (Tupaia belangeri; male = 12 and female = 12; average age, 1 year±3 months; average BW, 123.9±19.19 g) were purchased from the Kunming Institute of Zoology, Chinese Academy of Sciences. The tree shrews were reared in a single cage (35 cm × 25 cm × 30 cm, length, width, height, respectively), with a rest room set up in each cage for sleeping (15 cm × 12 cm ×12 cm, length, width, height, respectively). The tree shrews were housed under controlled conditions of temperature (23~25°C), relative humidity (40~50%) and a light/dark cycle of 12 hours.

### Experimental protocol

**Fig 1:**
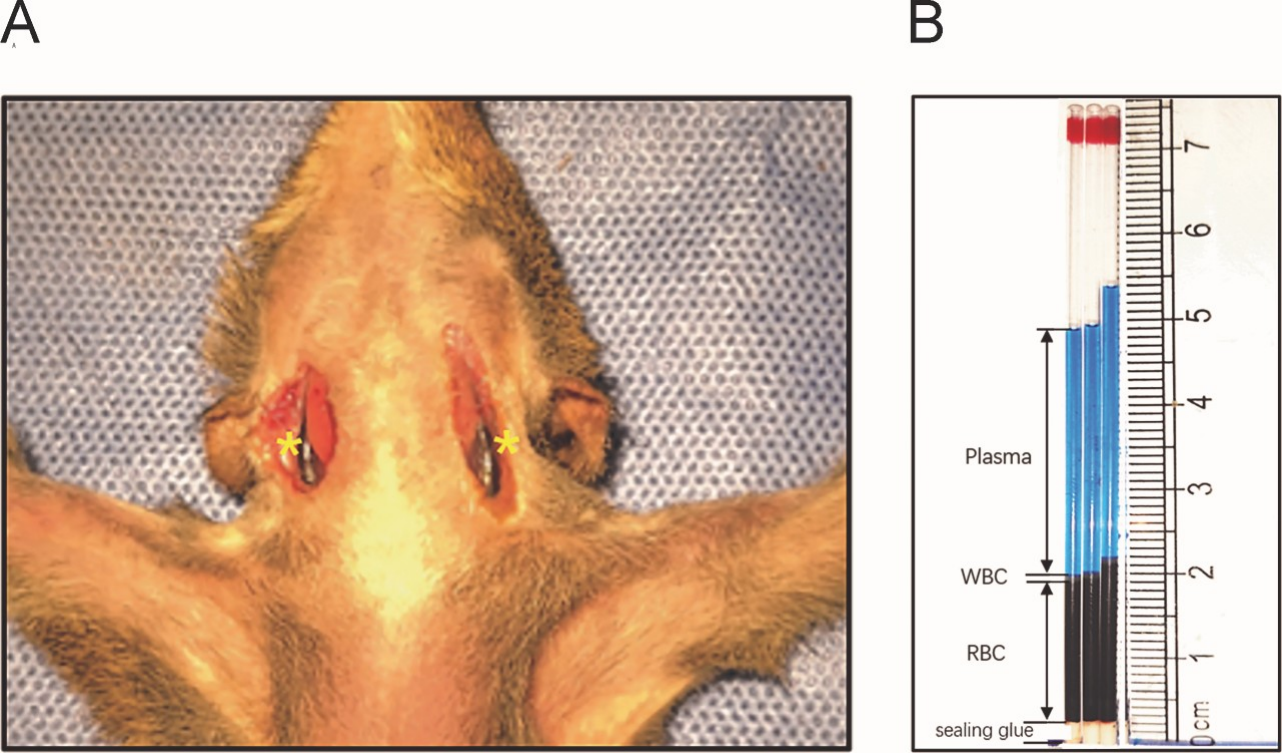
A: Jugular vein of tree shrew (Shown in yellow *); B: Blood component of tree shrew (after injection of Evans Blue). WBC: White blood cell; RBC: Red blood cell.

The tree shrews were fed with standard pellet diet and the feed was changed every morning to keep fresh. Animals were fasted for 12 hours before the start of the experiment to prevent excessive blood lipids from increasing plasma turbidity. The tree shrews were anesthetized with 1% sodium pentobarbital (Solarbio Science & Technology Company, Beijing, China) at 60mg/kg, and the abdomen was fixed upward on the dissection table. An incision was made parallel to the midline at a site approximately 1.0 cm away from the left and right sides of the neck midline. The skin was separated layer by layer under the microscope (BX51 WI, Olympus, Tokyo, Japan) to avoid damaging the blood vessels, until the bilateral jugular veins were exposed (Fig 1A). After the initial blood collection (100ul, time zero, T0), 100 ul of 2% Evans Blue dye (Sigma, Munich, Germany) was slowly injected into the right jugular vein using a 1ml syringe with a 30G needle, and another 1ml syringe with a 30G needle was inserted into the left jugular vein for blood sampling. The syringe was rinsed with 300 ul normal saline to ensure that all dye solutions were injected into the tree shrew. Separate injection and sampling containers were used to avoid contamination of the sample with residual dyes.

At 2, 4, and 6 minutes (T2, T4, T6) after the injection, blood sampling was performed and the extracted volume (ranging between 100–150 ul) carefully recorded. The blood samples were centrifuged at 1900 g for 30 minutes. The plasma supernatant was transferred to a second tube and re-centrifuged at 14000 g for 1 minute. The plasma supernatant was transferred to a clean tube and diluted 20 times with normal saline. All the dilutions were then analyzed with a spectrophotometer (NanoDrop 2000, Thermo Fisher Scientific, Pittsburgh, PA) at 620 nm. The standard curve was drawn with 9 points of equal dilution of Evans blue solution, and the two-compartment model (calculation method in S1 File) used to calculate the plasma volume.

After the collection of blood samples required for calculating the plasma volume was completed, 50 ul blood samples were collected separately from caudal vein, femoral vein, jugular vein, and heart. Then the blood of each site was transferred immediately into three glass tubes with anticoagulant (Thermo Fisher Scientific, Pittsburgh, PA) using the capillary siphon method. The length of the blood column accounted for 2/3 to 3/4 of the total length of the glass tube to avoid the formation of air bubbles. The blood-sucking end of capillary glass tube were inserted into the sealant seal vertically to a sealant column between 0.2–0.7 cm. The capillary tubes (with the sealing end outward) were centrifuged at 12000 g for 5 minutes. The length of the RBC layer and the whole blood layer were measured using a scale, and the ratio calculated as the hematocrit of this site (Fig 1B). As much blood as possible (about 5–8 ml in total, depending on the BW) was collected from each site (caudal vein, femoral vein, jugular vein, and heart) and mixed in the 15 ml centrifuge tube of heparin sodium anticoagulant. The hematocrit of mixed blood was measured by microhematocrit method to obtain the mean body hematocrit using the formula:

$$Blood\;volume=\frac{plasma\;volume}{1-mean\;body\;hematocrit}$$

### Statistical analysis

Data were assessed for normality using Shapiro-Wilk normality test. When data did not conform to normal distribution, the Mann–Whitney U-test was applied to perform two groups comparisons, and the nonparametric Kruskal–Wallis test was applied for the multiple groups. Gender differences (male vs female) and Weight differences (<120g vs. >120g) in each parameter were analyzed using the Unpaired Student t test. One-way ANOVA with Tukey’s multiple comparison test was used to determine whether the means of hematocrit and F factor in different parts of the body (mean body, cardiac, jugular vein, femoral vein and tail vein) were significantly different from each other. Data were analyzed using Graph Pad Prism, version 8.0.2 (Graph Pad Software, San Diego, CA, USA). P < 0.05 was set as a statistically significant level. Linear fitting was completed using fitting tools in OriginPro, version 2019b (Origin Labs, Farmington, ME, USA).

## Results

**Fig 2:**
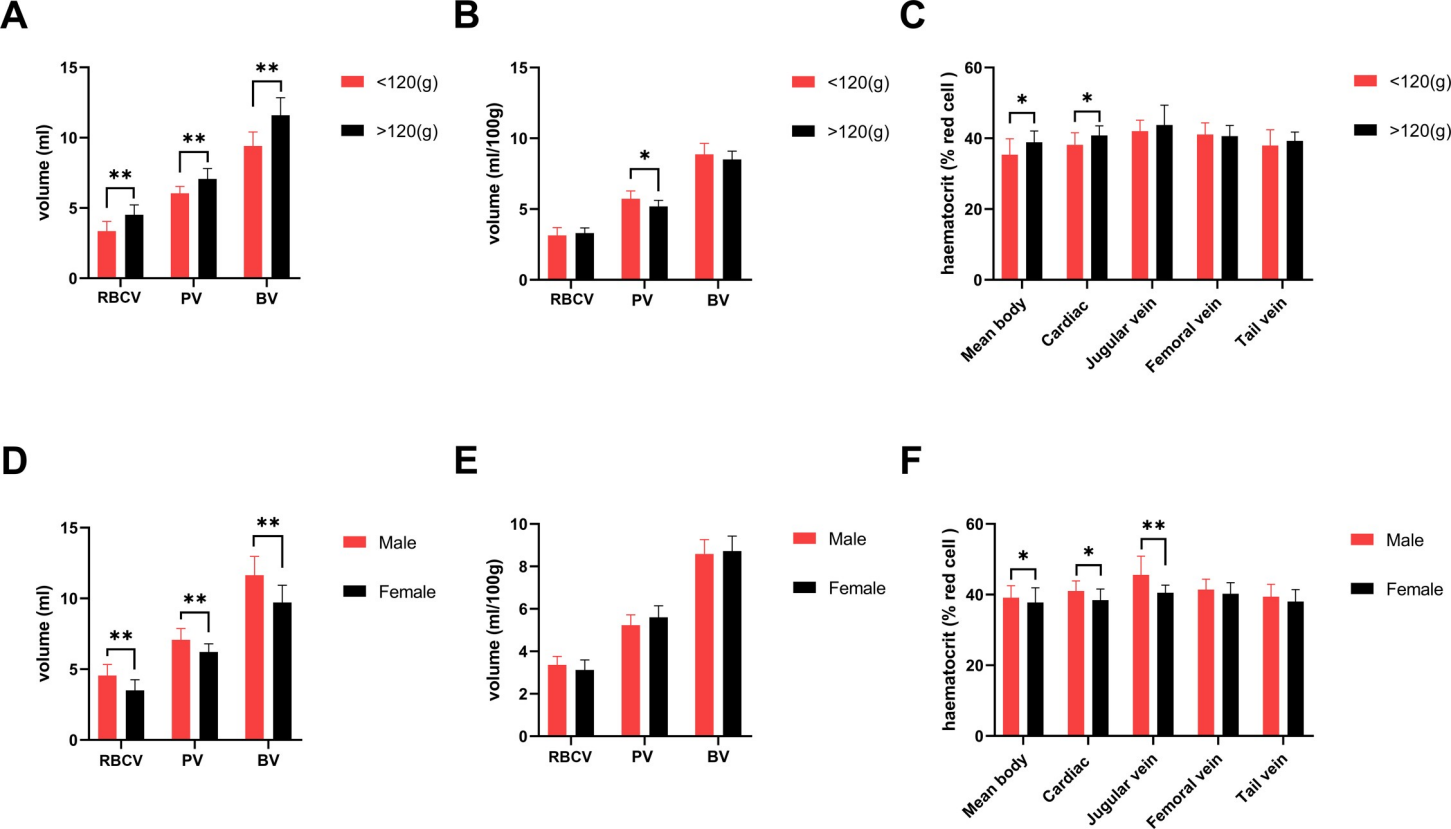
Blood component parameters of tree shrews with different body weight and gender. RBCV: Red blood cell volume; PV: Plasma volume; BV: Blood volume. **A:** A comparison of red blood cell volume, plasma volume, and blood volume of tree shrew by body weight; **B:** A comparison of red blood cell volume, plasma volume and blood volume for per 100g of tree shrew by body weight. **C:** A comparison of hematocrit in different sites of the tree shrew by body weight. **D:** A comparison of red blood cell volume, plasma volume and blood volume of tree shrew by gender. **E:** A comparison of red blood cell volume, plasma volume and blood volume per 100g body weight by gender. **F:** A comparison of hematocrit in different blood sampling sites by gender. *: p < 0.05;* *: p < 0.01.

The two-compartment model used in this study has good applicability to the observed values (estimated error (ERR) less than 1%). The volumes of plasma, RBC, blood, and BW are presented in Table 1. The mean volumes per 100g BW of plasma, RBC and total blood were 5.42±0.543, 3.24±0.445, 8.66±0.680ml, respectively. The volumes of plasma, RBC and total blood were higher in tree shrews with BW more than 120g than those with BW less than 120g (p <0.01, Fig 2A). However, for the mean volume plasma, RBC and total blood per 100g BW, a difference was observed in only the mean plasma volume between the two groups with the BW < 120g having higher volume (p <0.05, Fig 2B). The plasma volume, RBC volume and blood volume of tree shrews were higher in males than in females (p <0.01, Fig 2D). However, there was no difference in the average volumes per 100g body weight of mean plasma, RBC and total blood volumes between the males and females tree shrews (Fig 2E).

**Table 1:** Body weight and blood volumes in tree shrew.

	Weight			Sex			Total
	<120 (g)	>120 (g)	P	Male	Female	P	
Body weight (g)	106.2±9.147	136.5±13.38	0.000	136.1±17.3	111.6±11.95	0.001	123.9±19.19
Plasma volume (ml)	6.05±0.482	7.07±0.726	0.001	7.08±0.795	6.22±0.577	0.006	6.65±0.810
Red Cell Blood volume (ml)	3.35±0.696	4.52±0.703	0.001	4.56±0.768	3.51±0.749	0.002	4.03±0.904
Blood volume (ml)	9.40±1.005	11.59±1.254	0.000	11.64±1.334	9.72±1.210	0.001	10.68±1.583
Plasma volume (ml/100g)	5.73±0.551	5.20±0.427	0.014	5.23±0.492	5.60±0.548	0.096	5.42±0.543
RBC volume (ml/100g)	3.14±0.546	3.31±0.363	0.379	3.36±0.400	3.12±0.473	0.196	3.24±0.445
Blood volume (ml/100g)	8.87±0.772	8.51±0.588	0.200	8.59±0.675	8.72±0.708	0.646	8.66±0.680
ERR(%)	0.02±0.007	0.01±0.004		0.01±0.039	0.02±0.007		0.01±0.006
Kout(%/min)	4.34±1.246	3.86±1.941		4.04±1.812	4.09±1.6		4.06±1.672
Kin(%/min)	1.65±0.344	1.62±0.346		1.55±0.287	1.72±0.376		1.63±0.338
n	10	14		12	12		24

Values are expressed as mean ± standard deviation

RBC: Red blood cell

ERR: Estimated error

Kout: Outflow rate of dye from the distribution compartment (dye leak from plasma)

Kin: Inflow and outflow rate of dye between the injection compartment and the distribution compartment.

Mean (n = 24) body hematocrit, cardiac hematocrit, jugular vein hematocrit, femoral vein hematocrit, and tail vein hematocrit obtained by bloodletting were 37.43 ± 4.096, 39.72 ± 3.219, 43.04 ± 4.717, 40.84 ± 3.041, 38.71 ± 3.442% respectively. The results of ANOVA indicated that blood collection sites significantly affected hematocrit (F = 7.666, p < 0.01). Analysis between groups with Tukey’s multiple comparisons test showed the mean body hematocrit was significantly different from the hematocrit from specific sites of the body, such as jugular and femoral vein (P < 0.05). Among the hematocrit of these 5 different sites, BW > 120 g have higher values of mean body hematocrit and cardiac hematocrit than BW < 120 g tree shrews. The values of mean body, cardiac and jugular vein hematocrits of male were higher than those of female tree shrews (p < 0.05, Fig 2C and 2E). The F factor of heart blood (F cardiac), jugular vein blood (F jugular vein), femoral vein blood (F femoral vein) and tail vein blood (F tail vein) of tree shrews were 0.94 ± 0.072, 0.88 ± 0.118, 0.92 ± 0.111 and 0.97 ± 0.117 respectively (n = 24, Table 2). Analyses of variance revealed a significant differences in F factors of different blood sites (F = 3.345, p < 0.05). However, there was no significant difference in the blood F factors of all the sites of tree shrews between different weights groups and sex (p >0.05).

**Table 2:** Haematocrit and F factor in different anatomical region.

	Weight			Sex			Total
	<120 (g)	>120 (g)	P	Male	Female	P	
Mean body haematocrit (%)	35.36±4.504	38.90±3.165	0.034	39.10±3.423	37.75±4.156	0.043	37.43±4.096
Cardiac haematocrit (%)	38.19±3.366	40.81±2.723	0.047	41.01±2.866	38.42±3.131	0.046	39.72±3.219
Jugular vein haematocrit (%)	42.04±3.085	43.76±5.609	0.391	45.57±5.296	40.52±2.144	0.006	43.04±4.717
Femoral vein haematocrit (%)	41.09±3.265	40.65±2.982	0.739	41.44±2.957	40.23±3.131	0.344	40.84±3.041
Tail vein haematocrit (%)	37.95±4.479	39.26±2.512	0.373	39.41±3.492	38.02±3.395	0.335	38.71±3.442
F cardiac haematocrit	0.93±0.080	0.95±0.066	0.336	0.96±0.083	0.93±0.059	0.378	0.94±0.072
F jugular vein haematocrit	0.84±0.113	0.90±0.120	0.251	0.87±0.124	0.89±0.117	0.745	0.88±0.118
F femoral vein haematocrit	0.87±0.128	0.96±0.085	0.055	0.95±0.104	0.90±0.118	0.292	0.92±0.111
F tail vein haematocrit	0.94±0.145	0.99±0.090	0.285	1.00±0.100	0.95±0.131	0.299	0.97±0.117

Values are expressed as mean ± standard deviation

F cardiac = Mean body hematocrit/Cardiac hematocrit

F jugular vein = Mean body hematocrit/Jugular vein hematocrit

F femoral vein = Mean body hematocrit/Femoral vein hematocrit

F tail vein = Mean body hematocrit/Tail vein hematocrit

**Fig 3:**
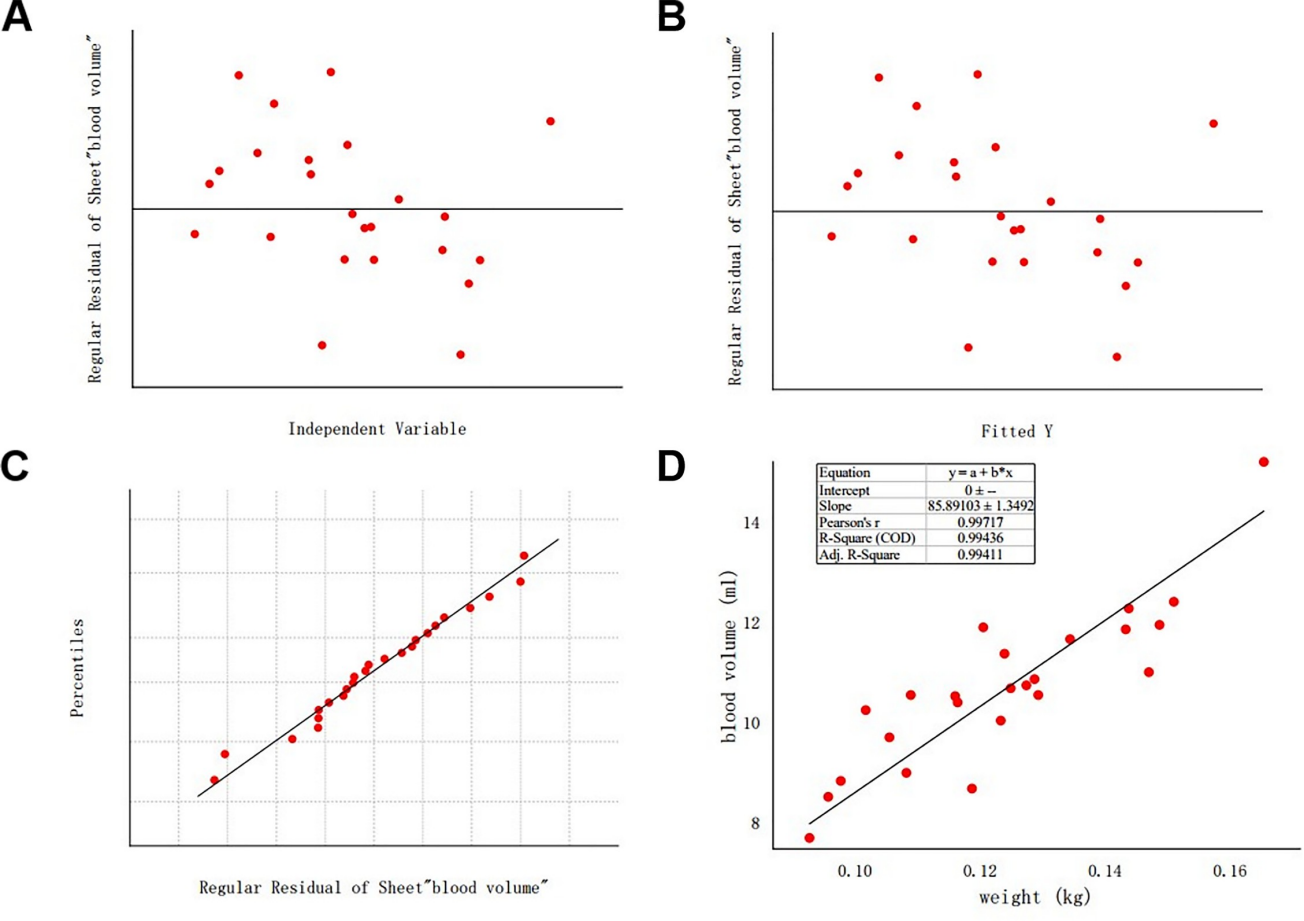
Fitting of body weight and blood volume of tree shrew. **A** and **B** are residual scatter plots of independent variables and predicted values; **C** shows the p-p diagram of the residual value; **D** shows graphical presentation of linear fitting between body weight and blood volume.

The effect of BW on blood volume of tree shrews was analyzed with the linear fitting tool of origin software. The standardized residual scatter diagram and the p-p diagram showed that the residual variances were homogeneous and normally distributed (Fig 3A–3C). The fitting equation was blood volume (ml) = 85.89103× BW (kg). The effect of BW on blood volume was statistically significant (F = 4052.20954, P < 0.001). BW accounted for 99.436% of the variation in blood volume, with a significant effect (Adj. R^2^ = 99.411%).

## Discussion

In many cases, blood samples need to be collected in animal experiments because they can provide valuable biological information. The measurement of blood volume in the animal is an important premise to determine volume of blood collected to avoid adverse effects (e.g. animals stress response and even hemorrhagic shock and death.) on experimental results [26]. Furthermore, blood volume of the conventionally used laboratory animals has been reported for a long time (e.g. mouse 9.63 ± 2.7 ml/100g [27]; rat 6.40±0.52 ml/100g [28]; rabbit 5.42±0.13 ml/100g [29]). This study has measured, for the first time, the blood volume (8.66±0.68 ml/100g) of tree shrews through a two-compartment model. It provides guidance for the formulation of welfare measures related to blood collection in tree shrews and widens the application of tree shrews in the field of biomedical research. There is similar to be observed in rats [9], the RBC volume per 100g BW was roughly constant, but the plasma volume per 100g BW decreased with the increase of BW in tree shrews. This suggested that the increase of plasma volume in small animals might be related to hemodynamic factors of small blood vessels [28]. In addition, blood volume have been shown to vary between rat strains and developmental stage [9,12], and It is also worth exploring in tree shrew.

The F factor is essential for calibration of hematocrit of venous blood [30].For instance, the F factor values of many other laboratory animals have been measured, for example: rat (mean F factor value, 0.98), rabbit (mean F factor value, 0.85), monkey (mean F factor value, 0.83) [7]. In our study, we investigated the F factor in the common blood collection site of tree shrews, and further explored its relationship with BW and sex. It was found that BW and sex affected mean body hematocrit, cardiac hematocrit and jugular vein hematocrit of tree shrews but F factor was stable and was not affected. This result verified the necessity and reliability of F factor calibration. The tail vein hematocrit of the tree shrew was close to the mean body hematocrit, and could be attributed to the relative difficulty of collecting blood from the tail vein. The skin of the tail vein of the tree shrew is black unlike the tail of rats which can easily identify the location of the tail vein. Furthermore, the tail vein of the tree shrew is finer, and the blood pressure decreases after anesthesia [31]. For these reasons, it is usually difficult to collect pure venous blood and in most cases the blood collected is mixed arteriovenous blood. Therefore, we do not recommend collecting blood from the tail vein of tree shrews.

For purposes of ensuring the reliability of the experimental results, the total amount of blood sampling for measuring plasma volume was only 400 ul. The total amount of blood sampled for measuring hematocrit of common blood collection sites was only 200 ul, and 300 ul of liquid was added after dye injection. Therefore, the plasma volume and hematocrit measured in this study avoided the adverse effects of hemodynamic changes due to excessive blood loss. Previously conducted studies believed that there was about 4% trapped plasma in RBCs after centrifugation, so the actual hematocrit was the reading value × 0.96 [7,12]. This theory was proposed based on Wintrobe method. However, this method has been gradually eliminated because it takes long time and involves large amount of blood. In this experiment, microhematocrit method recommended by WHO was used, which needed less blood and was easy to operate. High-speed centrifugation was also used to greatly reduce the trapped material, which is almost negligible. We did not therefore correct the hematocrit in our experiment.

In short, we calculated the parameters related to the blood volume of tree shrews. We also provided the venous blood F factor of the commonly used blood sampling sites and the formula for estimating the blood volume of tree shrews using their body weight. The BW and sex affected the volumes of plasma, RBC and blood, and mean body, cardiac and jugular vein hematocrits of tree shrews. We hope that our research can provide reference for the related research on blood volume of tree shrews in the future.

## Supporting information

S1 File Supplemental data. (PDF)

S2 File (XLSX)
