# Supplementary material for: Plasma volume, cell volume, total blood volume and F factor in the tree shrew
Source: PLoS One. 2020 Sep 3;15(9):e0234835. doi: 10.1371/journal.pone.0234835 (PMC7470369; doi:10.1371/journal.pone.0234835)
Supplement: S1 File — (PDF) [file pone.0234835.s001.pdf]

Richalet et al. Modeling the Evans blue dilution method for the measurement of plasma volume: a new optimized method.

.

Supplemental data

```
// Method for estimating Plasma Volume with a dye dilution technique (Evans blue)
 // Based on a two-compartment model with injection compartment and distribution (plasma) compartment
 // Jean-Paul Richalet, University Paris 13, September 2017 – Scilab- 6.0.0
 clear
 // Entry of all measured parameters
 rat=input("animal # ","s");
 m=4;
 Mi=input("mass of dye injected (mg)= ? ");
 Vi=input('injected volume (ml)= ? ');
 tm(1)=input('sampling time 1 (s)= ? ' )+1; // zero but should be 1 for matrix handling
 V1=input('withdrawn volume 1 (ml)= ? ' );
 Cpo(1)=input('dye concentration 1 (mg/ml)= ? ');
 tm(2)=input('sampling time 2 (s)= ? ' );
 V2=input('withdrawn volume 2 (ml)= ? ');
 Cpo(2)=input('dye concentration 2 (mg/ml)= ? ');
 tm(3)=input('sampling time 3 (s)= ? ' );
 V3=input('withdrawn volume 2 (ml)= ? ' );
 Cpo(3)=input('dye concentration 3 (mg/ml)= ? ');
 tm(4)=input('sampling time 4 (s)= ? ' );
 V4=input('withdrawn volume 4 (ml)= ? ' );
 Cpo(4)=input('dye concentration 4 (mg/ml)= ? ');
 Hct=input("hematocrit (p.cent)= ? ");
 V1=V1*(1-Hct/100);V2=V2*(1-Hct/100);V3=V3*(1-Hct/100);V4=V4*(1-Hct/100);//blood volume to plasma
volume sampled
 ym=Cpo;wm =ones(m,1);
 function y=yth(t, x)
    y=x(1)*exp(-x(3)*t)-x(1)*exp(-(x(2)+x(3))*t) // Integration of the two differential equation for two-
compartments model
 endfunction
 // Estimation of Vp and Kout by the Log linear method
 x=[0,0,0];
 y=[0,0,0];
 for i=1:1:m-1
    x(i)=tm(i+1)
    y(i)=ym(i+1)
 end
 y=log(y);
 [a,b,sig]=reglin(x,y);
 Cpomaxreg=exp(b); // Concentration at time=0
 Vpreg=Mi/Cpomaxreg+V1+V2+V3+V4-Vi;// Calculation of Plasma volume, taking into account volume of
plasma injected or withdrawn
 Koutreg=-a*100*60;// Escape rate of dye by the log linear method in %/min
Kin0=ym(2)/(Cpomaxreg*tm(2));Kout0=-a; // Estimation of Kin and Kout for initializing the identification
process
 x0= [Cpomaxreg;Kin0;Kout0];// initial values are taken from the log linear extrapolation
 function e=myfun(x, tm, ym, wm) // calculation of function e to be minimized (sum of square differences)
   e = wm.*( yth(tm, x) - ym )
 endfunction
 [f,xopt, gopt] = leastsq(list(myfun,tm,ym,wm),x0) // process of least square identification
 // plotting the results
 tt = linspace(0,1.1*max(tm),100)';
 yy = yth(tt, xopt);
 scf();
```

```
plot(tm, ym, "kx")
plot(tt, yy, "k-")
legend(["measure points", "fitted curve"]);
a=get("current axes");
t=a.title;
t.font_size=5;
x_label=a.x_label;
x_label.font_size=5;
y_label=a.y_label;
y_label.font_size=5;
titre="Identification of plasma volume with Evans blue. Animal#"+string(rat);
xtitle(titre,"time(s)","dye concentration (mg/mL)")
Vp=Mi/xopt(1)+V1+V2+V3+V4-Vi;// Calculating Vp, taking into account volume of plasma injected or withdrawn
Kout=xopt(3)*100*60;Kin=xopt(2)*100;// Calculating escape rate of dye (Kout, in %/min) and injection rate (Kin, in %/s)
ERR=f/sum(ym.*ym)*100;// estimation of error, as percentage of sum of square values
// printing the results
p1="Kout (%/min)= ";p2="Vp (mL)= ";p3="ERR (%)= ";
format(5);
disp("Animal # :"+rat)
disp("Results with two-compartment model")
disp(p3+string(ERR),p1+string(Kout),p2+string(Vp))
disp("Kin (%/s)= "+string(Kin))
disp("Results with log linear regression :")
format(5);
disp("Kout (%/min) = "+string(Koutreg),"Vp reg (mL) = "+string(Vpreg))
//Example : animal # : HS007, mass of dye injected=200, injected volume=0.55, sampling time 1 = 0,
//withdrawn volume 1 = 0.76, dye concentration 1 = 0, sampling time 2 = 128, withdrawn volume 2 = 0.74,
//dye concentration 2 = 18.209, sampling time 3 = 244, withdrawn volume 3 = 0.67, dye concentration 3 = 13.503
//sampling time 4 = 360, withdrawn volume 4 = 0.69, dye concentration 4 = 11.845, hematocrit = 60
//%
//Animal # : HS007
//Results with two-compartment model
//Vp (mL) = 9.06
//Kout (%/min)= 12.2
//ERR (%)= 0.25
//Kin (%/s)= 3.06
//Results with log linear regression
//Vp reg (mL)= 9.50
//Kout reg (%/min)= 11.1

// in our study, Mi=2; Vi=0.1; tm(1)=0+1; V1=0.1; V2=0.1; V3=0.1; V4=0.1; Cpo(1)= 0.00;
// The value of  tm(2); tm(3); tm(4);Cpo(2) ; Cpo(3); Cpo(4); Hct are in Main Document Table1
```
